# Ethyl Pyruvate Ameliorates Hepatic Ischemia-Reperfusion Injury by Inhibiting Intrinsic Pathway of Apoptosis and Autophagy

**DOI:** 10.1155/2013/461536

**Published:** 2013-12-25

**Authors:** Miao Shen, Jie Lu, Weiqi Dai, Fan Wang, Ling Xu, Kan Chen, Lei He, Ping Cheng, Yan Zhang, Chengfen Wang, Dong Wu, Jing Yang, Rong Zhu, Huawei Zhang, Yinqun Zhou, Chuanyong Guo

**Affiliations:** Department of Gastroenterology, The Tenth People's Hospital of Tongji University, Shanghai 200072, China

## Abstract

*Background*. Hepatic ischemia-reperfusion (I/R) injury is a pivotal clinical problem occurring in many clinical conditions such as transplantation, trauma, and hepatic failure after hemorrhagic shock. Apoptosis and autophagy have been shown to contribute to cell death in hepatic I/R injury. Ethyl pyruvate, a stable and simple lipophilic ester, has been shown to have anti-inflammatory properties. In this study, the purpose is to explore both the effect of ethyl pyruvate on hepatic I/R injury and regulation of intrinsic pathway of apoptosis and autophagy. *Methods*. Three doses of ethyl pyruvate (20 mg/kg, 40 mg/kg, and 80 mg/kg) were administered 1 h before a model of segmental (70%) hepatic warm ischemia was established in Balb/c mice. All serum and liver tissues were obtained at three different time points (4 h, 8 h, and 16 h). *Results*. Alanine aminotransferase (ALT), aspartate aminotransferase (AST), and pathological features were significantly ameliorated by ethyl pyruvate (80 mg/kg). The expression of Bcl-2, Bax, Beclin-1, and LC3, which play an important role in the regulation of intrinsic pathway of apoptosis and autophagy, was also obviously decreased by ethyl pyruvate (80 mg/kg). Furthermore, ethyl pyruvate inhibited the HMGB1/TLR4/ NF-**κ**b axis and the release of cytokines (TNF-**α** and IL-6). *Conclusion*. Our results showed that ethyl pyruvate might attenuate to hepatic I/R injury by inhibiting intrinsic pathway of apoptosis and autophagy, mediated partly through downregulation of HMGB1/TLR4/ NF-**κ**b axis and the competitive interaction with Beclin-1 of HMGB1.

## 1. Introduction

Hepatic ischemia-reperfusion (I/R) injury is predominantly encountered during hemorrhagic shock, hepatolobectomy, hepatic transplantation, and trauma, which may cause hepatocyte necrosis, liver disfunction, and even liver failure [1, 2]. Hepatic I/R injury results in the activation of Kupffer cells, neutrophils, and platelets, with subsequent inflammation and cell injury. The damage of hepatic sinusoidal endothelial cells contributes to microcirculatory disturbances that eventually exacerbate hepatic I/R injury, creating a vicious cycle. Hepatic I/R injury also leads to the upregulation of various proinflammatory cytokines, such as interleukin 2 (IL-2), IL-6, IL-1, tumor necrosis factor *α* (TNF-*α*), and high mobility group box 1 (HMGB1) [3–5]. And hepatic I/R injury has been a general and severe disease in our daily clinical work; thus, the protection of liver against I/R injury has become increasingly important.

There exist complicated mechanisms in the occurrence and development of hepatic I/R injury. It has been demonstrated that the activation of Kupffer cells, production of cytokines, cell adhesion factor, and reactive oxygen species (ROS) play a pivotal role in the pathogenesis of hepatic I/R injury [6, 7]. The main pathological changes of hepatic I/R injury are the neutrophil infiltration and liver cell death caused by diverse factors. According to the current study, liver cell death of hepatic I/R injury mainly exhibits as necrosis and apoptosis [8]. Necrosis, a kind of nonprogrammed cell death responding to external injury, act as organelle swelling and membrane breakdown, following inflammatory reactions. Apoptosis, conversely, named type I programmed cell death, was a genetically determined process that started with the activation of cell surface molecule by external factor and, ensuing the expression of related gene, finally resulted in breaking cell into small-membrane-wrapped vesicles, namely, apoptotic bodies [9, 10]. There are several signal pathways that work in the regulation of apoptosis and are mainly divided into two interconnected mechanisms: caspase-dependent classical apoptosis and caspase-independent programmed form of cell death. As a classical pathway, caspase-dependent apoptosis is initiated either by extrinsic or intrinsic factors. And transmembrane receptors such as TNF/TNFR and Fas/FasL contribute to the origination of extrinsic pathway by receiving external signals, further activating caspase 8 and other downstream caspases [11, 12]. On the other hand, Bcl-2 family is considered to have an important role in the intrinsic pathway, also called mitochondrial pathway. The Bcl-2 family consists of proapoptotic and antiapoptotic members. The representative apoptosis-inhibiting genes are Bcl-2 and Bcl-xl, and the others are Bax and Bad. It has been reported that the balance between Bax and Bcl-2 proteins determines the possibility of cells to survive or undergo apoptosis after a certain stimulus or injury [13–15].

Recently, autophagy, as a new manner of cell death, has attracted scientists' attention worldwide. It includes macroautophagy, microautophagy, and chaperone-mediated autophagy [16]. Among these, macroautophagy is generally known as the formation of autophagosomes, which encircle deserted cellular components or impaired organelles and carry them to lysosomes to form autolysosomes [17]. In a certain extent, autophagy keeps the stabilization of cell by recycling new cell components [18, 19]. However, when beyond this range, autophagy will finally result in the cell death with the overweening accumulation of autophagosomes, especially under the continuous stimulation of starvation, hypoxia, and inflammation [20–25]. The process of autophagy is also regulated by related gene. Microtubule-associated protein light chain 3 (LC3) was generally recognized as a marker to monitor autophagy. When autophagy is upregulated, a cleaved cytosolic form of LC3 (LC3-I) is integrated to phosphatidylethanolamine to form LC3-phosphatidylethanolamine (LC3-II), which exclusively is expressed on the autophagosome. In addition, Beclin-1 is also a pivotal regulatory factor in the process of autophagy. Several proteins could combine with it and influence the function, such as Bcl-2 and HMGB1 [26]. Except for its own regulatory mechanisms, autophagy is simultaneously controlled by upstream signal pathways, for example, PI3 K/Akt, mitogen-activated protein kinase, and mammalian target of rapamycin [27, 28]. Gujral et al. supposed that inhibitors of caspases could attenuate to hepatic I/R injury if there existed apoptosis only; however, there was no significant effect according to their results [29]. Therefore, it is believable that other mechanisms of cell death also existed in the process of hepatic I/R injury. And this fact was further demonstrated afterwards, especially autophagy related cell death in hepatic I/R injury [30, 31].

In brief, there exist several mechanisms of cell death in hepatic I/R injury, including apoptosis, necrosis, and autophagy. Therefore, we suspect that if we could find a new drug or method to interfere with or obstruct the process of cell death, the hepatic I/R injury may be ameliorated significantly. Ethyl pyruvate, a stable and simple lipophilic ester that originates from the endogenous metabolite pyruvate, has been shown to protect against inflammation and attenuates organ dysfunction in several animal models of clinical illnesses, such as burn injury, severe sepsis, and acute pancreatitis [32, 33]. Recently, the effects of ethyl pyruvate on I/R injury have also been demonstrated by scientists worldwide. In 2012, Hu et al. reported that ethyl pyruvate reduces myocardial I/R injury by inhibiting the expression of HMGB1 protein in rats [34], and Shen et al. demonstrated that ethyl pyruvate ameliorates hypoxic-ischemic brain injury via anticell-death and anti-inflammatory mechanisms [35]. Tsung et al. also showed that ethyl pyruvate protects against hepatic I/R injury by reducing hepatic necrosis and apoptosis. However, they just reveal that ethyl pyruvate might ameliorate hepatic I/R injury through inhibiting the external pathway of apoptosis and necrosis [36]. Whether ethyl pyruvate could affect cell death caused by the intrinsic pathway of apoptosis and autophagy was not explored, and the internal relationship was also not clarified; therefore, we set this experiment to further explore the effect of ethyl pyruvate on hepatic I/R injury and possible mechanisms.

## 2. Materials and Methods

### 2.1. Reagents

Ethyl pyruvate was purchased from Sigma-Aldrich (Saint Louis, MO, USA). The antibodies used in this study included those directed against HMGB1 (Epitomics, CA), TLR4 (BioLegend, CA), IL6 (Proteintech, CA), TNF-*α* (Santa Cruz, CA), Bcl-2 (cell signal technology, USA), Bax (cell signal technology, USA), LC3 (cell signal technology, USA), Beclin-1 (cell signal technology, USA), inhibitor proteins of NF-*κ*B *α* (I*κ*B *α*) (cell signal technology, USA), inhibitor proteins of NF-*κ*B *β* (I*κ*B *β*) (cell signal technology, USA), and NF-*κ*B (Proteintech, CA).

### 2.2. Animal Preparation

Male Balb/c mice (6–8 weeks old, 23 ± 2 g) were purchased from Shanghai Laboratory Animal Co. Ltd. Shanghai, China. The mice were raised in a clean room maintained at 24 ± 2°C under a 12 h : 12 h light : dark cycle, with free access to food and water. All animal experiments were approved by the Animal Care and Use Committee of Shanghai Tongji University.

### 2.3. Model Establishment and Experimental Design

A model of segmental (70%) hepatic warm ischemia was established using a previously reported method [37]. Male Balb/c mice fasted for 16–24 h and were placed on a sterile experimental table after they had been anesthetized with 1.25% Nembutal (Saint Louis, MO, USA). A midline laparotomy was then performed in all animals. All the structures in the portal triad (hepatic artery, portal vein, and bile duct) to the left and median liver lobes were occluded for 45 min with a metal microvascular clamp. Continuous reperfusion was achieved by loosening the clamps. After reperfusion, the abdominal cavity was closed with surgical thread, and the mice were placed in a warm environment until they a woke.

The mice were allocated randomly to one of three groups as follows.

 Group I (saline only): 18 mice were injected with saline via the tail vein 1 h before laparotomy. The subsequent laparotomy was performed without I/R.
 Group II (saline + I/R): 18 mice were injected with saline via the tail vein 1 h before they were laparotomized and then subjected to I/R for 45 min.
 Group III (EP + I/R): 54 mice were injected with ethyl pyruvate averagely (20 mg/kg, 40 mg/kg, and 80 mg/kg) via the tail vein 1 h before they were laparotomized and then subjected to I/R for 45 min.

Six mice were randomly selected from group I and group II, 18 mice were selected from group III (6 mice in every dose of ethyl pyruvate), and all selected mice were killed 4 h, 8 h, and 16 h after hepatic I/R. All sera and liver tissues (median and left lobes) were collected and stored for further analysis.

### 2.4. Measurement of Liver Enzymes

Sera were collected by centrifuging all blood samples at 2000 rpm for 10 min. The serum levels of alanine aminotransferase (ALT) and aspartate aminotransferase (AST) were measured with an automated chemistry analyzer (Olympus AU1000, Japan) to evaluate the hepatic parenchymal damage.

### 2.5. Histopathology

When the mice were killed, their liver tissues (median and left lobes) were collected, incubated in 4% paraformaldehyde, and embedded in paraffin wax according to the traditional method. Sections (4 *μ*m thick) were cut and stained with hematoxylin-eosin (H&E) for observation under a light microscope.

### 2.6. Immunohistochemical Staining

The sections were prepared by heating at 67°C for 20 min. After they were dewaxed in dimethylbenzene for 10 min, the sections were dehydrated in a graded series of alcohol. Antigen retrieval was then performed by microwaving the samples in citrate buffer for four cycles (in one cycle, the citrate buffer was heated to boiling and cooled for 5 min). Treatment with 3% H_2_O_2_ for 20 min at room temperature blocked endogenous peroxidase activity. The cell membranes were then ruptured with 0.3% Triton X-100 (Saint Louis, MO, USA) for 30 min at 37°C. Nonspecific proteins were blocked with 5% bovine serum albumin (BSA) for 30 min. The sections were finally incubated overnight with anti-HMGB1 and anti-NF-*κ*B, anti-IL-6, anti-TNF-*α*, anti-Bcl-2, anti-Bax, anti-LC3, anti-Beclin-1, and anti-TLR4 (the membrane was not ruptured with 0.3% Triton X-100) antibodies at 4°C. On day 2, the sections were washed with phosphate-buffered saline (PBS) and treated immediately with the secondary antibody (1 : 500 in PBS) for another 30 min. The antibody was then visualized with a diaminobenzidine (DAB) kit and the specimens were observed under a light microscope.

### 2.7. Immunofluorescence

Fresh liver tissues collected from the mice were fixed in 4% paraformaldehyde on ice for 1 h. The fixed liver tissues were washed three times with PBS for 5 min on ice before they were dehydrated overnight in 30% sucrose (dissolved in PBS) at 4°C. The tissues were infiltrated with OCT (SAKURA, USA) for 2 h on day 2 and then frozen and stored at −80°C. Sections (5 *μ*m) were cut with a freezing microtome and stored at −20°C. Before analysis, the prepared sections were dried at room temperature for 5 min, after which the OCT was dissolved in PBS for 5 min. The cell membranes were ruptured with 0.2% Triton X-100 at room temperature for 20 min. Nonspecific antigen binding sites were blocked with 5% BSA and the sections were then incubated overnight with HMGB1 (1 : 1000) at 4°C. After the samples were incubated with anti-rabbit antibody for 30 min on day 2, the cell nuclei were stained with 2-(4-amidinophenyl)-6-indolecarbamidine dihydrochloride (DAPI) (1 : 1000). All sections were observed with fluorescence microscopy.

### 2.8. Western Blotting

Proteins were extracted from the tissues stored at −80°C for western blotting analysis. The proteins were then incubated in boiling water for 10 min, separated by sodium dodecyl sulfate polyacrylamide gel electrophoresis (SDS-PAGE), transferred to a Polyvinylidene Fluoride Membrane (PVDF membrane), blocked with 5% milk for 1 h, and incubated overnight with anti-HMGB1, TLR4, NF-*κ*B, I*κ*B *α*, I*κ*B *β*, TNF-*α*, IL-6, LC3, Beclin-1, Bax, and Bcl-2 antibodies at 4°C. After the samples were incubated with a secondary antibody for 30 min at 37°C, the signal was detected with the Odyssey Two-Color Infrared Laser Imaging System (LI-COR Biosciences, Lincoln, Neb).

### 2.9. TUNEL Staining

The sections were deparaffinized and rehydrated. After that, TUNEL staining was performed according to the instructions for the TUNEL assay kit. Then the sections were counterstained with hematoxylin. Finally, total hepatocytes and TUNEL-positive cells were observed under light microscopy.

### 2.10. Electron Microscopy

To observe the autophagic vesicles, liver tissues were fixed with 3% glutaraldehyde in 0.2% mol/L sodium cacodylate. And before being dissected, the specimens were treated with 1% osmium tetroxide for 1 hour. Finally, the cells were observed under the electron microscopy (JEM1230, Japan).

### 2.11. SYBR Green Real-Time RT-PCR

Total RNA was isolated from the collected liver tissues using TRIzol Reagent (Takara Japan, Shiga, Japan). The RNA was reverse transcribed into cDNA according to the manufacturer's instructions (Takara). Equal quantities of cDNA were continuously amplified by PCR in a 10 *μ*L reaction volume. The primers used for RT-PCR (see Table 1).

### 2.12. Statistical Analysis

All results are expressed as the mean ± SD. Comparison between groups was performed using Student's *t*-test and one-way analysis of variance. In all comparisons, *P* < 0.05 was considered statistically significant. All statistical analyses were performed using SPSS 13.0 for Windows.

## 3. Results

### 3.1. Ethyl Pyruvate Pretreatment Ameliorates Hepatic I/R Injury

**Figure 1:**
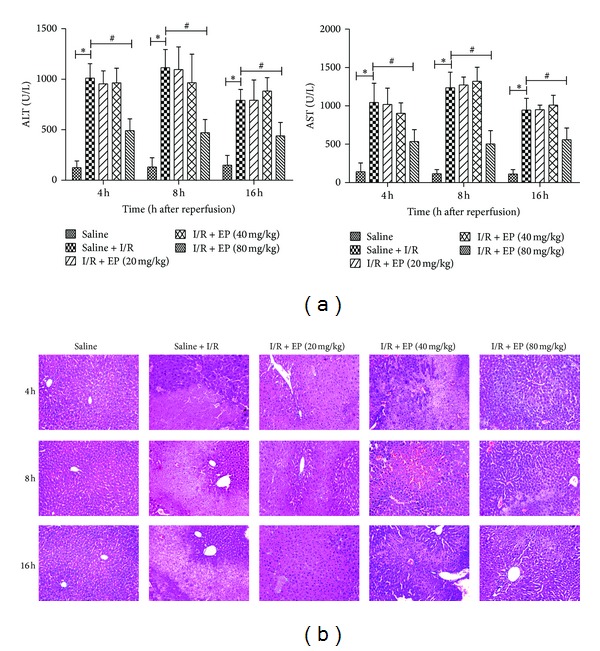
Effect of ethyl pyruvate on hepatic ischemia-reperfusion injury. The I/R and sham-operated mice were pretreated with ethyl pyruvate (20 mg/kg, 40 mg/kg, and 80 mg/kg) or saline. Mice were sacrificed 4 h, 8 h, and 16 h after reperfusion. The serum ALT and AST levels were assayed, shown in (a). Data represent means (SD) (*n* = 6 mice per time point per group). *P < 0.05 for saline VS saline + I/R, ^#^ *P* < 0.05 for saline + I/R VS I/R + EP (80 mg/kg). Representative hematoxylin and eosin (H&E) stained sections of liver are shown in (b). Original magnifications: ×200.

To assess the effects of ethyl pyruvate on hepatic I/R injury, the levels of ALT and AST in sera acquired from each group of mice were measured. As shown in Figure 1(a), the levels of ALT and AST increased clearly in the saline + I/R group compared with the saline group at 4 h, 8 h, and 16 h (*P* < 0.05). Conversely, there were significant reductions in the levels of ALT and AST after ethyl pyruvate (80 mg/kg) treatment at all three time points (*P* < 0.05). However, the doses of 20 and 40 mg/kg work ineffectively (*P* > 0.05). The pathological features of the liver tissues from the three groups after H&E staining are also shown in Figure 1(b). The structures of the liver tissues were completely maintained and remained ordered in the saline-only group, whereas a disordered lobular structure, marked hepatocyte necrosis, and polymorphonuclear cell infiltration were observed in the saline + I/R group at 4 h, 8 h, and 16 h. However, the administration of ethyl pyruvate (80 mg/kg) clearly reduced all the pathological features apparent in the saline + I/R group. And the pathological change of ethyl pyruvate (20 mg/kg and 40 mg/kg) was not obviously compared to group II (saline + I/R).

### 3.2. Ethyl Pyruvate Reduces Bax/Bcl-2 Ratio and Amount of Apoptotic Cells

**Figure 2:**
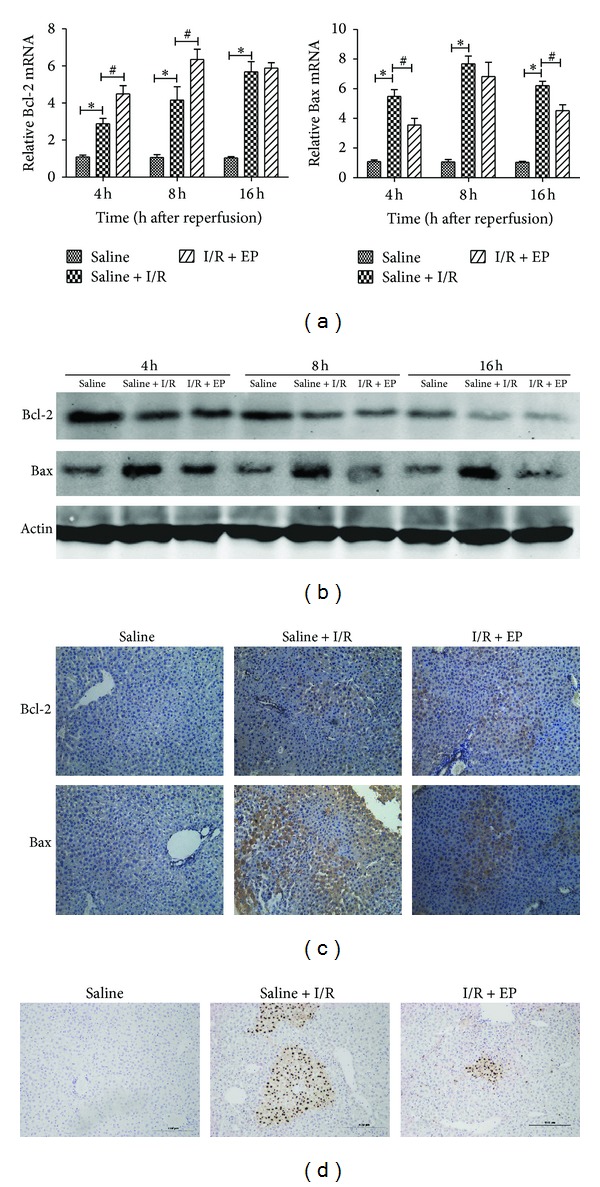
Effect of ethyl pyruvate on regulation of apoptosis. (a) The expression of Bcl-2 and Bax on cDNA level was detected by real-time PCR (_ _*P < 0.05 for saline VS saline + I/R, _ _^#^ *P* < 0.05 for saline + I/R VS I/R + EP (80 mg/kg). (b) The expression of Bcl-2 and Bax on protein level was detected by western blot. (c) Immunohistochemistry staining showed the expression of Bcl-2 and Bax protein in liver tissue at 8 h. Original magnifications: ×200. (d) TUNEL staining showed the apoptotic cells in three groups at 8 h. Original magnifications: ×100.

As previously mentioned, apoptosis resulted in the cell death of hepatic I/R injury, causing hepatic disfunction. Therefore, to explore the potential protective mechanism of ethyl pyruvate against hepatic I/R injury, we measured the changes in Bcl-2 and Bax at the cDNA and protein levels in the three groups. In view of the ineffective doses of 20 mg/kg and 40 mg/kg, we investigated the possible mechanism with the dose of 80 mg/kg in the next experiments. The expression of Bcl-2 and Bax cDNAs was detected with real-time PCR, as shown in Figure 2(a). It is clear that ethyl pyruvate pretreatment significantly reduced the expression of Bax at 4 h and 16 h and increased the expression of Bcl-2 at 4 h and 8 h. Ethyl pyruvate also reduced the expression of Bax at the protein level at all three time points, and the expression of Bcl-2 increased mainly at 4 h and 8 h with ethyl pyruvate treatment (Figure 2(b)). In addition, there existed a similar result showed in immunohistochemistry at 8 h (Figure 2(c)). The apoptotic cells were detected by TUNEL staining, as shown in Figure 2(d), and a number of TUNEL-positive cells were observed in the saline + I/R group, however the amount in I/R + EP group was markedly decreased.

### 3.3. Ethyl Pyruvate Inhibits the Expression of Beclin-1 and LC3 and Decreases the Amount of Autophagosomes

**Figure 3:**
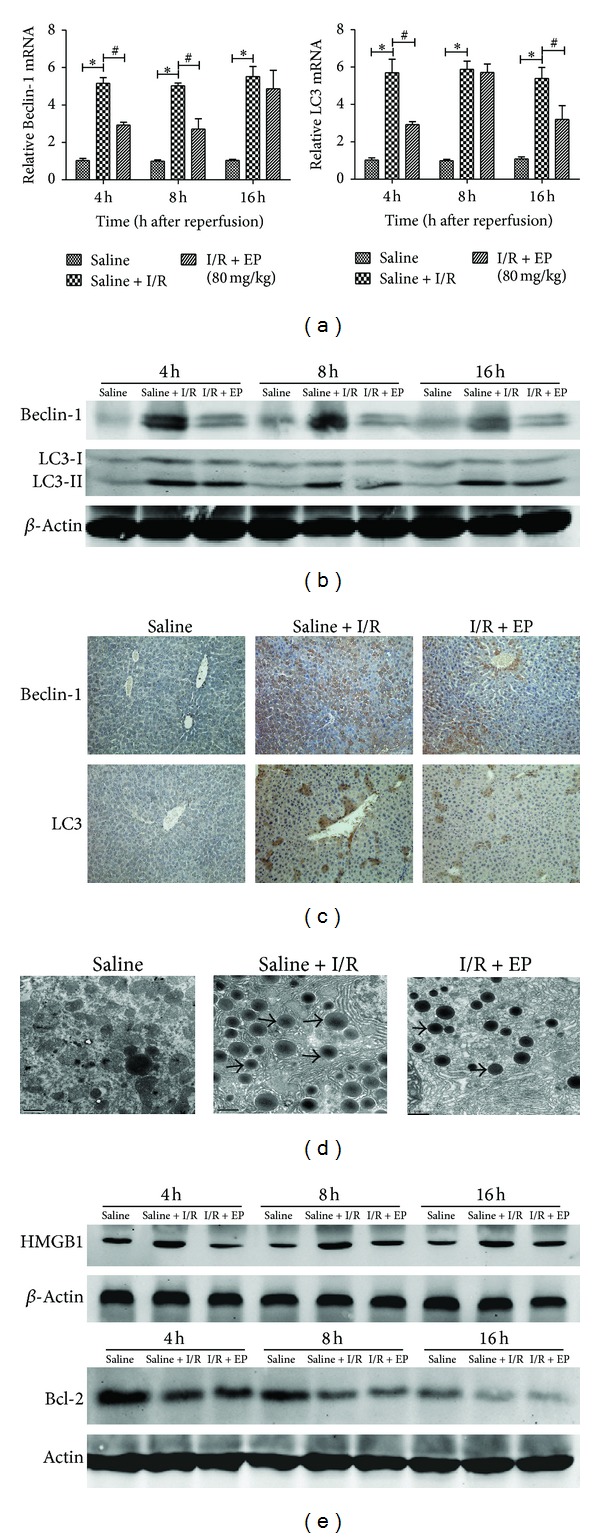
Effect of ethyl pyruvate on regulation of autophagy. (a) The expression of Beclin-1 and LC3 on cDNA level was detected by real-time PCR (_ _*P < 0.05 for saline VS saline + I/R, _ _^#^ *P* < 0.05 for saline + I/R VS I/R + EP (80 mg/kg)). (b) The expression of Beclin-1 and LC3 on protein level was detected by western blot. (c) Immunohistochemistry staining showed the expression of Beclin-1 and LC3 protein in liver tissue at 8 h. Original magnifications: ×200. (d) Electron microscopy showed the ultrastructure and autophagosomes (“→” indicated the autophagosomes) at 8 h. Original magnifications: ×10000. (e) Effect of ethyl pyruvate on the expression of HMGB1 and Bcl-2 was detected by western blot at three time points.

It is well known that Beclin-1 and LC3 play an important role in the regulation of autophagy. Therefore, we examined the changes of Beclin-1 and LC3 in both cDNA and protein levels separately by real-time PCR and western blot. The results indicated that the levels of Beclin-1 and LC3 had a significant reduction with ethyl pyruvate treatment compared to saline + I/R group no matter in cDNA or protein level (Figures 3(a) and 3(b)). And this result is concordant with the change detected by immuohistochemistry at 8 h (Figure 3(c)). On the other hand, the formation of autophagosomes is a pivotal process in the occurrence of autophagy; hence, we further used the technology of electron microscopy to observe the ultrastructure of hepatic cell. It was shown that a significant ultrastructural morphological change was found in saline + I/R group (8 h), such as mitochondrial swelling, crest damage, and increase in lysosomal and autophagosome. However, with ethyl pyruvate treatment, liver nuclear chromatin was more homogenous, the structure could still be integral, with less amount of lysosomal and autophagosome (Figure 3(d)).

### 3.4. Ethyl Pyruvate Inhibits the Expression and Translocation of HMGB1

**Figure 4:**
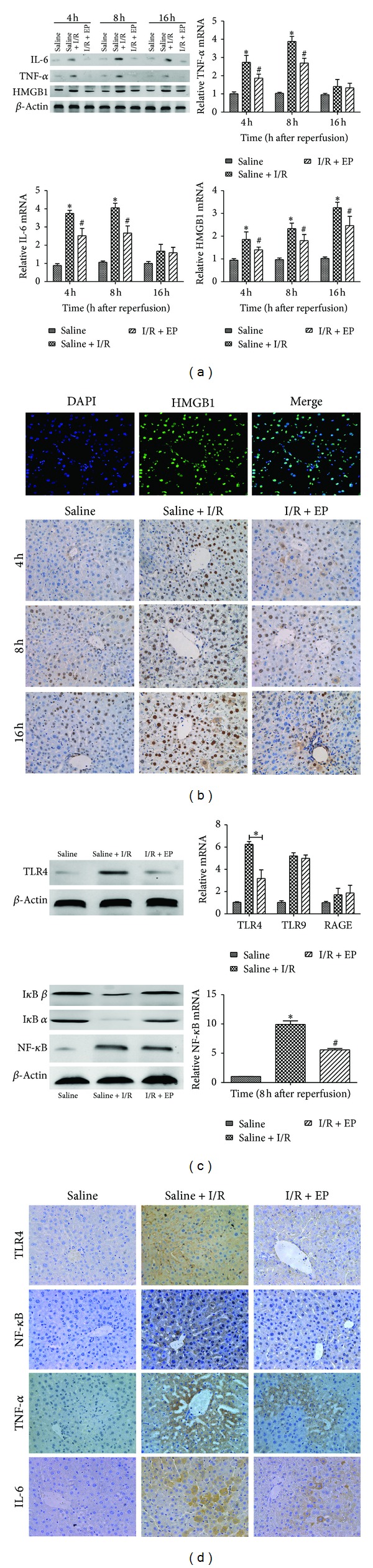
(a) The expression of HMGB1, TNF-*α*, and IL-6 on cDNA level was detected by real-time PCR (_ _*P < 0.05 for saline VS saline + I/R, _ _^#^ *P* < 0.05 for saline + I/R VS I/R + EP (80 mg/kg)). And the expression of HMGB1, TNF-*α*, and IL-6 on protein level was detected by western blot. (b) HMGB1 was located in nucleus of normal liver tissue by immunofluorescence (original magnifications: ×200). The expression of HMGB1 in liver tissue of different groups was shown by immunohistochemistry (original magnifications: ×400). (c) The expression of TLR4 and NF-*κ*B level was detected by real-time PCR (_ _*P < 0.05 for saline VS saline + I/R, _ _^#^ *P* < 0.05 for saline + I/R VS I/R + EP (80 mg/kg)). And the expression of TLR4, I*κ*B *α*, I*κ*B *β*, and NF-*κ*B on protein level was detected by western blot. (d) Immunohistochemistry staining showed the expression of TLR4, NF-*κ*B, IL-6, and TNF-*α* protein in the liver tissue (original magnifications: ×400).

It is well known that HMGB1 is more strongly expressed after the administration of hepatic I/R and migrates to the cytoplasm where it acts. Therefore, we detected the level of HMGB1 by real-time PCR and western blot (Figure 4(a)). The results showed that there existed a reduction of expression of HMGB1 with ethyl pyruvate treatment compared to saline + I/R group. Meanwhile, we found that HMGB1 was mostly located in nuclei by immunofluorescence in normal tissue and examined the changes in HMGB1 in the liver tissues collected from the three experimental groups using immunohistochemistry. As shown in Figure 4(b), HMGB1 was clearly located in the nuclei and was expressed at low levels in the saline group. However, after hepatic I/R was induced, HMGB1 was expressed more strongly in the nuclei and had partly translocated to the cytoplasm. In contrast, pretreatment with ethyl pyruvate significantly reduced the expression of HMGB1, in both the nuclei and cytoplasm.

### 3.5. Ethyl Pyruvate Reduces the Expression of TLR4, NF-*κ*B, TNF-*α*, and IL-6 and Inhibits the Degradation of I*κ*B *α* and I*κ*B *β*

TLR4, TLR9, and RAGE, as the main receptors of HMGB1, have been shown to contribute to the activity of the HMGB1 signaling pathway in various disease models. Here, we firstly detected the expression of TLR4, TLR9, and RAGE on cDNA level 8 h after reperfusion. Results showed that the expression of TLR4 decreased in I/R + EP group compared to saline + I/R group at 8 h (Figure 4(c)). And there was no a significant change in the level of TLR9 and RAGE. The same result was demonstrated by western blot. NF-*κ*B signal pathway is activated mainly through the degradation of I*κ*B, which plays a pivotal role in promoting inflammation, and TNF-*α* and IL-6 are the main cytokines to initiate the process of apoptosis. Here we found that the administration of ethyl pyruvate obviously blocked the degradation of I*κ*B *α* and I*κ*B *β* detected by western blotting. And results also showed that the expression of NF-*κ*B, TNF-*α*, and IL-6 was only significantly increased in saline + I/R group, as demonstrated by real-time PCR and western blotting. However, ethyl pyruvate treatment resulted in the downregulation of NF-*κ*B, TNF-*α*, and IL-6 (Figures 4(a) and 4(c)). In addition, we deeply investigated the location and expression of TLR4, NF-*κ*B, TNF-*α*, and IL-6 by immunohistochemical staining. It was markedly to observe that all of these four indexes were expressed more strongly in saline + I/R group than in the saline group at 8 h. In contrast, after ethyl pyruvate treatment, there was a significant reduction in the specific areas in which they were expressed (Figure 4(d)).

## 4. Discussion

Hepatic I/R injury is a common clinical problem, occurring during traumatic shock, organ transplantation, and surgical operations. Serracino-Inglott et al. have demonstrated that the morbidity associated with liver transplantation and major hepatic resection is closely associated with I/R injury [38]. Hence, hepatic I/R injury has already attracted the attention of scientists worldwide. The exploration of more effective drugs and instruments is urgently required.

The protective effects of ethyl pyruvate, a stable lipophilic ester, have already been demonstrated in multifarious inflammatory injuries, including sepsis and hemorrhagic shock [39]. Here, we have shown that ethyl pyruvate attenuates hepatic I/R injury and that the histopathological changes caused by I/R, such as cellular necrosis, neutrophil infiltration, and cellular swelling, are clearly ameliorated by ethyl pyruvate, which are consistent with changes in ALT and AST (Figure 1). It is well known that Bax promotes intrinsic apoptosis by forming oligomers in the mitochondrial outer membrane, participating in the release of apoptogenic molecules; oppositely, Bcl-2 inhibits mitochondrial apoptosis by blocking the release and oligomerization of Bax. The balance between Bax and Bcl-2 proteins has also been linked with the induction of apoptosis in cell death in kidney, heart, and brain after I/R [40–42]. And our results showed that the balance could not be maintained because of the increase of Bax and decrease of Bcl-2 in saline + I/R group, which finally resulted in the cell death. However, with ethyl pyruvate treatment, the number of TUNEL-positive hepatic cells had a significant decrease; meanwhile, the balance between Bax and Bcl-2 trended to normal, with the upregulation of Bcl-2 and downregulation of Bax (Figure 2). Hence, we supposed that ethyl pyruvate ameliorated cell death in hepatic I/R injury by inhibiting the intrinsic pathway of apoptosis.

Autophagy, type II programmed cell death, has been reported to participate in causing kinds of organs' I/R injury, such as lung, heart, kidney, and liver, through form autophagosomes, degrade organelles and essential compositions. It provides a new target for us to protect against I/R injury. Therefore, we investigated the effect of ethyl pyruvate on regulation of autophagy in hepatic I/R injury by detecting the expression of Beclin-1 and LC3, two pivotal regulatory genes in autophagy. We clearly found that both Beclin-1 and LC3 expressed less with ethyl pyruvate treatment compared to saline + I/R group in all three time points (Figure 3). And it has been demonstrated that the upregulation of Beclin-1 promotes the process of autophagy; meanwhile, LC3-II is the only protein expressed on autophagosome. Based on this, our results showed that ethyl pyruvate might attenuate to hepatic I/R injury by downregulating the process of autophagy. And we further confirmed our thinking by observing the reduction of autophagosomes in ethyl pyruvate treatment group under the electron microscope.

**Figure 5:**
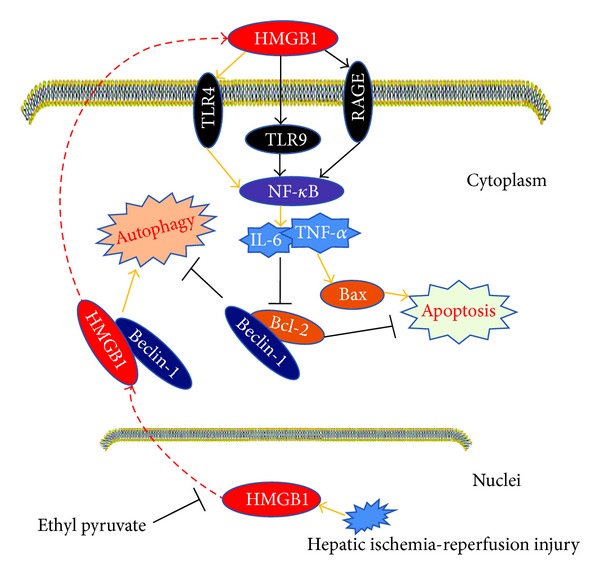
In the condition of hepatic ischemia-reperfusion injury, HMGB1 is predominantly released by stressed hepatocytes, which actively secrete HMGB1 into the circulation. After release, HMGB1, as a proinflammatory cytokine, combined with TLR4 was expressed on the surface of hepatocytes or other nonparenchymal cells. NF-*κ*B signal pathway is further activated to promote cytokine release, such as TNF-*α* and IL-6. Under the stimulation of TNF-*α* and IL-6, intrinsic pathway of apoptosis is initiated, exhibited as the upregulation of Bax and downregulation of Bcl-2. On the other hand, with the translocation of HMGB1 into cytoplasm from nuclei, HMGB1 competitively combined with Beclin-1 to promote the level of autophagy through representing the site of Bcl-2, which can maintain the inactive status of autophagy. Ethyl pyruvate successfully inhibits the expression and translocation of HMGB1 in stressed cells under the condition of hepatic I/R injury and further downregulates intrinsic pathway of apoptosis and autophagy to ameliorate hepatic I/R injury.

Here, we had shown that ethyl pyruvate might ameliorate hepatic I/R injury by decreasing both apoptosis and autophagy. And, in the next experiment, we tried to explore possible mechanisms included in this process. Recently, HMGB1, a DNA-binding protein, has been recognized as a late inflammatory cytokine during sepsis that is actively released by monocytes and macrophages [43]. In addition to its function in sepsis, the activity of HMGB1 in I/R injury has also been explored by scientists worldwide. In 2008, Martin et al. reported that HMGB1 plays a pivotal role in cardiac I/R injury [44], and similar studies of other organs have been undertaken, including the kidney, brain, and intestine [45]. In these tissues, HMGB1 is released into the cytoplasm after I/R injury and promotes inflammation by integrating the activities of members of the toll-like receptor (TLR) family and the receptor for advanced glycation end products (RAGE) [46]. And some reports had indicated that ethyl pyruvate could decrease the expression of HMGB1 in I/R injury [46], not including hepatic I/R injury. And as showed in our results, ethyl pyruvate also had the ability to inhibit the expression and translocation of HMGB1 in hepatic I/R injury (Figure 4(b)). Whereas how did ethyl pyruvate regulate autophagy by decreasing the expression of HMGB1? It is well known that Beclin1 is an essential autophagic protein, having an important role in the initiation of autophagy. It includes three identified structural domains, named BH3 domain, central coiled-coil domain, and evolutionarily conserved domain. And antiapoptotic Bcl-2 family members, mainly Bcl-2 protein, interact with BH3 domain to maintain the inactive status of autophagy. Relatively, if this site is competitively occupied by other molecules, the Beclin-1-regulated pathway partly activates and promotes the process of autophagy. Tang et al. demonstrated that HMGB1 was competed with Bcl-2 for interaction with Beclin-1, finally downregulating autophagosome formation in some cell lines, such as mouse Panc02, human HCT116, and mouse RAW264.7 [47]. Hence, we supposed that ethyl pyruvate might inhibit the process of autophagy in hepatic I/R injury partly because of the interaction between HMGB1 and Beclin-1. On the other hand, as set forth, with the increase of Bcl-2 after ethyl pyruvate treatment, the combination between Beclin-1 and Bcl-2 more closely also resulted in the downregulation of autophagy (Figures 3(e) and 5).

Apoptosis can be regulated by cytokines such as TNF-*α* and IL-6 which can be enhanced by TLR4 and its related pathway, such as NF-*κ*B pathway during I/R injury. In addition, the receptors of HMGB1 are mostly members of the toll-like receptor (TLR) family and the receptor for advanced glycation end products (RAGE). These receptors contribute to the activation of the mitogen-activated protein kinases (MAPKs) and NF-*κ*B pathway to facilitate the extracellular function of HMGB1 [48]. Importantly, NF-*κ*B acts as a transcription factor that mediates the cellular responses to a wide variety of extracellular stress stimuli [49]. It also triggers the upregulation of cytokines such as TNF-*α* and IL-6 to initiate the inflammatory responses and process of apoptosis. Firstly, we investigated which receptor played the major role to receive the signal from HMGB1 under the effect of ethyl pyruvate in our model. The results of real-time PCR showed that TLR4 decreased significantly with ethyl pyruvate treatment compared to the change of TLR9 and RAGE. And the change of TLR4 was further confirmed by western blot and immunohistochemical staining. Secondly, results showed that the degradation of I*κ*B *α* and I*κ*B *β* was significantly blocked by ethyl pyruvate, and the expression of NF-*κ*B was also decreased after ethyl pyruvate treatment, which uniformly demonstrated that ethyl pyruvate could downregulate NF-*κ*B signal pathway in our model. Meanwhile, the expression of TNF-*α* and IL-6 was also decreased with ethyl pyruvate treatment in our model. Therefore, we suspected that there existed HMGB1/TLR4/NF-*κ*B axis to execute the antiapoptotic effect of ethyl pyruvate in hepatic I/R injury (Figure 5).

Finally, in our experiment, we demonstrated that ethyl pyruvate ameliorated hepatic I/R injury by inhibiting intrinsic pathway of apoptosis and autophagy. And ethyl pyruvate might decrease these two type programmed cell death (apoptosis and autophagy) separately through the downregulation of HMGB1/TLR4/NF-*κ*B axis and the competitive interaction of HMGB1 with Beclin1. Our results showed that apoptosis and autophagy could be critical therapeutic targets of hepatic I/R injury in clinic. In the future, ethyl pyruvate could be a fine choice to treat hepatic I/R injury.

## Figures and Tables

**Table 1:** 

Gene		Primer sequence (5′→3′)
TNF-*α*	Forward	CAGGCGGTGCCTATGTCTC
	Reverse	CGATCACCCCGAAGTTCAGTAG
TLR4	Forward	GCCTTTCAGGGAATTAAGCTCC
	Reverse	GATCAACCGATGGACGTGTAAA
IL-6	Forward	CTGCAAGAGACTTCCATCCAG
	Reverse	AGTGGTATAGACAGGTCTGTTGG
HMGB1	Forward	GCATCCTGGCTTATCCATTGG
	Reverse	GGCTGCTTGTCATCTGCTG
NF-*κ*B	Forward	ATGGCAGACGATGATCCCTAC
	Reverse	CGGATCGAAATCCCCTCTGTT
*β*-actin	Forward	GGCTGTATTCCCCTCCATCG
	Reverse	CCAGTTGGTAACAATGCCATGT
Bax	Forward	AGACAGGGGCCTTTTTGCTAC
	Reverse	AATTCGCCGGAGACACTCG
Bcl-2	Forward	GCTACCGTCGTGACTTCGC
	Reverse	CCCCACCGAACTCAAAGAAGG
LC3	Forward	GACCGCTGTAAGGAGGTGC
	Reverse	AGAAGCCGAAGGTTTCTTGGG
Beclin-1	Forward	ATGGAGGGGTCTAAGGCGTC
	Reverse	TGGGCTGTGGTAAGTAATGGA
TLR9	Forward	ATGGTTCTCCGTCGAAGGACT
	Reverse	GAGGCTTCAGCTCACAGGG
RAGE	Forward	TGGGAATTACTATGCGTGCAAA
	Reverse	TGGATCTCTCGCAGACTGTTC
