# Corrigendum to “Ethyl Pyruvate Ameliorates Hepatic Ischemia-Reperfusion Injury by Inhibiting Intrinsic Pathway of Apoptosis and Autophagy”

**DOI:** 10.1155/2016/5434275

**Published:** 2016-10-11

**Authors:** Miao Shen, Jie Lu, Weiqi Dai, Fan Wang, Ling Xu, Kan Chen, Lei He, Ping Cheng, Yan Zhang, Chengfen Wang, Dong Wu, Jing Yang, Rong Zhu, Huawei Zhang, Yingqun Zhou, Chuanyong Guo

**Affiliations:** Department of Gastroenterology, The Tenth People's Hospital of Tongji University, Shanghai 200072, China

In the article titled “Ethyl Pyruvate Ameliorates Hepatic Ischemia-Reperfusion Injury by Inhibiting Intrinsic Pathway of Apoptosis and Autophagy” [1], the name of the fifteenth author was given incorrectly as Yinqun Zhou. The author's name should have been written as Yingqun Zhou. The revised authors' list is shown above.

## References

[1] Shen M., Lu J., Dai W. (2013). Ethyl pyruvate ameliorates hepatic ischemia-reperfusion injury by inhibiting intrinsic pathway of apoptosis and autophagy. *Mediators of Inflammation*.

